# Multi‐omics integration reveals a nonlinear signature that precedes progression of lung fibrosis

**DOI:** 10.1002/cti2.1485

**Published:** 2024-01-24

**Authors:** Céline Pattaroni, Christina Begka, Bailey Cardwell, Jade Jaffar, Matthew Macowan, Nicola L Harris, Glen P Westall, Benjamin J Marsland

**Affiliations:** ^1^ Department of Immunology, School of Translational Medicine Monash University Melbourne VIC Australia; ^2^ Department of Respiratory Medicine Alfred Hospital Melbourne VIC Australia

**Keywords:** disease progression, lipidomics, metabolomics, multi‐omics, pulmonary fibrosis, transcriptomics

## Abstract

**Objectives:**

Idiopathic pulmonary fibrosis (IPF) is a devastating progressive interstitial lung disease with poor outcomes. While decades of research have shed light on pathophysiological mechanisms associated with the disease, our understanding of the early molecular events driving IPF and its progression is limited. With this study, we aimed to model the leading edge of fibrosis using a data‐driven approach.

**Methods:**

Multiple omics modalities (transcriptomics, metabolomics and lipidomics) of healthy and IPF lung explants representing different stages of fibrosis were combined using an unbiased approach. Multi‐Omics Factor Analysis of datasets revealed latent factors specifically linked with established fibrotic disease (Factor1) and disease progression (Factor2).

**Results:**

Features characterising Factor1 comprised well‐established hallmarks of fibrotic disease such as defects in surfactant, epithelial–mesenchymal transition, extracellular matrix deposition, mitochondrial dysfunction and purine metabolism. Comparatively, Factor2 identified a signature revealing a nonlinear trajectory towards disease progression. Molecular features characterising Factor2 included genes related to transcriptional regulation of cell differentiation, ciliogenesis and a subset of lipids from the endocannabinoid class. Machine learning models, trained upon the top transcriptomics features of each factor, accurately predicted disease status and progression when tested on two independent datasets.

**Conclusion:**

This multi‐omics integrative approach has revealed a unique signature which may represent the inflection point in disease progression, representing a promising avenue for the identification of therapeutic targets aimed at addressing the progressive nature of the disease.

## Graphical abstract

This study aimed to understand the early molecular factors driving idiopathic pulmonary fibrosis (IPF), a progressive lung disease, by combining various omics data from healthy and IPF lung explants. Multi‐omics data integration identified distinct molecular factors associated with established fibrotic disease and disease progression.

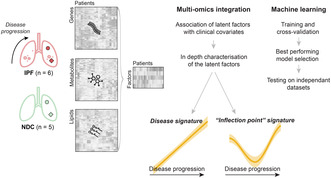

## Introduction

Idiopathic pulmonary fibrosis (IPF) is a severe lung disease of unknown aetiology characterised by progressive lung scarring. Although the pathophysiological mechanisms associated with the end stages of the disease are well established, the early events preceding the first symptoms and diagnosis of the disease remain unclear. The current postulate is that repeated micro‐injuries to the lung epithelium cause a dysfunctional phenotype leading to an aberrant wound healing process.^1^ While an aberrant epithelial phenotype is a central feature of the disease, a paradigm shift is needed in the way we consider IPF pathophysiology. Indeed, the mechanisms responsible for disease initiation (e.g. epithelial cell injury) may differ from those of disease progression. The complexity of the relationship between risk factors,^2^, ^3^ timing and activation of interconnected cellular pathways prompts us to move away from the current reductionist view of perceiving IPF pathogenesis as gradual and linear. In fact, it has recently been proposed that the aberrant activation of pathways in IPF might follow a chaotic dynamic pattern, with small changes in baseline conditions being sufficient to trigger a long‐term fibrotic response.^4^

High‐throughput molecular profiling technologies have allowed for unprecedented characterisation of human lung diseases using omics datasets, including transcriptomics, metabolomics and lipidomics, among others.^5^ Transcriptomics has particularly expanded our understanding of IPF pathophysiology by revealing gene expression patterns associated with fibrosis such as matrix metalloproteinases (MMPs), activation of (myo)fibroblasts, WNT signalling, host defence and metabolism.^6^ Meanwhile, metabolic characterisation of the lung microenvironment has shown alterations in amino acid (e.g. related to arginine)^7^, ^8^, ^9^ and glucose metabolism (e.g. shift towards lactic acid production), as well as changes in the beta‐oxidation pathway involving fatty acids (FA).^10^ Furthermore, lipids such as S1P and LPA may also contribute to IPF development.^11^ Multi‐omics studies aiming to simultaneously interrogate different layers of biomolecules have started to emerge, revealing the power of combining data modalities. However, compared with other respiratory diseases such as COPD/asthma or in the context of lung transplantation,^12^ integrative multi‐omics studies aiming to investigate IPF are scarce^13^ and often limited to animal models.^14^

In IPF, tissue remodelling typically begins at the basal posterior edges of the lungs and progresses apically, creating a unique opportunity to study disease progression by sampling different areas of the lung. Two studies^15^, ^16^ have revealed that macroscopically normal IPF tissue exhibits notable gene expression differences when compared to tissue from non‐IPF individuals. Both studies identified sets of genes with altered expression levels preceding the macroscopic evidence of fibrosis. Notably, these two previous investigations exclusively focused on gene expression. With this study, we aimed to model the leading edge of fibrosis using an unbiased multi‐omics data‐driven approach on structurally normal, pre‐fibrotic and fibrotic lung explant samples from IPF patients and non‐diseased controls (NDC).

## Results

### Idiopathic pulmonary fibrosis lung explants exhibit distinct molecular profiles when compared to controls

**Figure 1:**
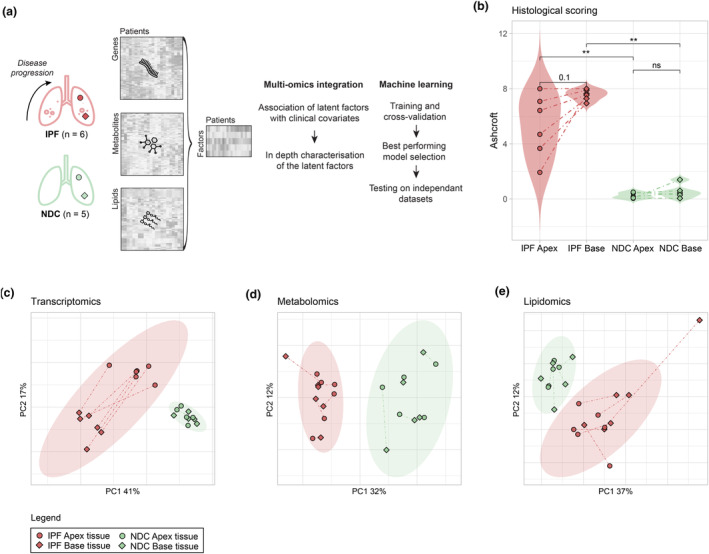
Study design and overall multi‐omics data variance. **(a)** Multi‐omics data (transcriptomics, metabolomics and lipidomics) from idiopathic pulmonary fibrosis (IPF) and non‐diseased controls (NDC) apex and base lung samples were integrated in an unsupervised fashion. Factors correlating with clinical metadata were investigated and used as input for machine learning and validation on an independent dataset. **(b)** Violin plots of lung tissues histological scoring (Ashcroft score) comparing disease groups and lung tissue location. Principal component analysis (PCA) of normalised transcriptomics **(c)**, metabolomics **(d)** and lipidomics **(e)** data with ellipses representing the 95% confidence intervals for each group. Colours are representative of disease status with dashed lines linking the apex and base samples obtained from the same individual. Sample sizes are 6 IPF apex, 6 IPF base, 5 NDC apex and 5 NDC base. Statistics represent the result of FDR‐corrected non‐parametric Wilcoxon Rank Sum testing with FDR < 0.05, < 0.01 and < 0.001 represented as *, ** and ***, respectively.

Three omics layers from lung explants taken from patients with IPF (*n* = 6) and NDC (*n* = 5) were simultaneously investigated. Patients' characteristics and key sample metadata are presented in Supplementary table 1. Transcriptomics (RNA sequencing), as well as untargeted metabolomics and lipidomics (LCMS) datasets, were generated for multi‐omics data integration and machine learning (Figure 1a). For each lung explant, a sample from the base (fibrotic, late‐stage of IPF) and a sample from the apex (pre‐fibrotic, early stage of IPF) were collected. In order to characterise the extent of fibrosis prior to molecular profiling, histological grading was performed using the Ashcroft scale (Figure 1b). Ashcroft scores from NDC samples were significantly lower than their topological IPF counterparts (Wilcoxon test FDR‐corrected *P*‐value < 0.01** for apex and < 0.01** for base), suggesting that fibrosis has already been initiated in IPF apices (representative histology slides presented in Supplementary figure 1). Differences in IPF apex and IPF base scores were variable (Wilcoxon test FDR‐corrected *P*‐value = 0.1). Principal component analysis (PCA) of normalised transcriptomics (Figure 1c), metabolomics (Figure 1d) and lipidomics (Figure 1e) completely separated IPF from NDC samples. Standard differential expression (transcriptomics) and intensity (metabolomics, lipidomics) testing on individual omics revealed marked differences with disease with 36%, 39% and 39% of the total features showing differential abundance in the transcriptomics, metabolomics and lipidomics datasets, respectively (Supplementary figure 2a, b). Differential gene expression patterns between the apex and base of IPF samples were observed (Supplementary figure 2c), a distinction not observed in NDC samples or metabolomics and lipidomics analysis. In addition, differential expression testing between apex samples from IPF against those from NDC revealed an upregulation of prototypical fibrotic remodelling genes, including MMPs and collagens, within the IPF apex (Supplementary figure 2d) indicating strong changes to cellular programmes before overt evidence of fibrosis. Given the marked differences observed by PCA and standard differential testing, we hypothesised that the integration of these multi‐omics datasets, combined with the topological sampling reflective of different disease stages, could reveal molecular drivers underpinning progression of pulmonary fibrosis.

### Multi‐omics analysis identifies factors correlating with established disease and progression

**Figure 2:**
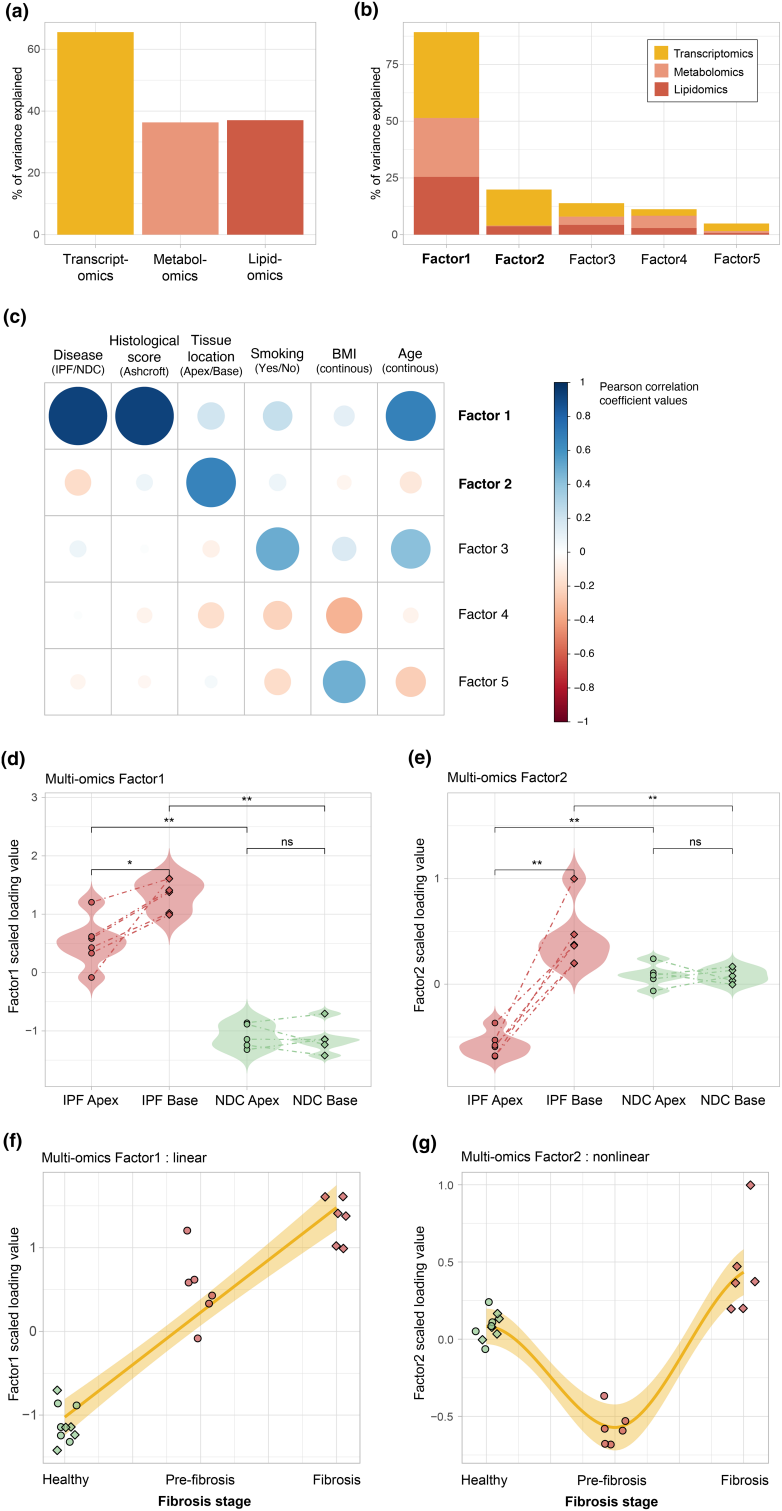
Unsupervised multi‐omics data integration using Multi‐Omics Factor Analysis (MOFA) and association with clinical covariates. **(a)** Cumulative proportions of total variance explained by each omics dataset. **(b)** Total variance explained by each omic view by individual factors. **(c)** Correlations between MOFA factors and clinical covariates with significant Pearson correlation coefficients (*P*‐value < 0.05) represented in a colour scale from red (negative correlations) to blue (positive correlations). **(d)** Violin plots of MOFA Factor1 values comparing disease groups and lung tissue location. **(e)** Corresponding violin plots for MOFA Factor2 values. **(f)** Factor1 values in relation to fibrosis stage proxy (non‐diseased controls (NDC), idiopathic pulmonary fibrosis (IPF) apex and IPF base) with lm fitting. **(g)** Factor2 values in relation to fibrosis stage proxy (NDC, IPF apex and IPF base) with loess curve fitting. Sample sizes are 6 IPF apex, 6 IPF base, 5 NDC apex and 5 NDC base. Statistics represent the result of FDR‐corrected non‐parametric Wilcoxon rank‐sum testing with FDR < 0.05, < 0.01 and < 0.001 represented as *, ** and ***, respectively.

We next aimed to integrate the different molecular layers characterising lung explant samples to uncover sources of variation that explain the progressive nature of the disease. We used Multi‐Omics Factor Analysis (MOFA),^17^ a statistical framework enabling the discovery of latent factors capturing the major sources of variation across omics datasets in an unsupervised fashion. MOFA identified five latent factors that cumulatively explained a significant proportion of variation in the transcriptomics (66%), metabolomics (36%) and lipidomics (37%) layers (Figure 2a). Factor1 explained most of the variance (*R* ^2^) and captured comparable proportions of variability across the different datasets (38% transcriptomics, 26% metabolomics and 25% lipidomics), which was also the case for Factor3 and 4 (Figure 2b). In contrast, Factor2 was mostly represented by the transcriptomics layer (16%) followed by lipidomics (4%). We next investigated the correlation between these factors and clinical variables and found that Factor1 was strongly associated with disease (Pearson correlation 0.93 *P*‐value < 0.001***), Ashcroft histological score (Pearson correlation 0.93 *P*‐value < 0.001***) and age (Pearson correlation 0.67 *P*‐value < 0.001***) (Figure 2c). Notably, Factor2 correlated with the lung tissue topographical location (apex versus base) (Pearson correlation 0.66 *P*‐value < 0.001***). Other significant correlations included smoking and BMI parameters with less pronounced correlation coefficients (Pearson correlation < 0.5). Differences in Factor1 values were further investigated by comparing disease and topological sampling subgroups. Factor1 completely separated samples from IPF and NDC (Wilcoxon test FDR‐corrected *P*‐value < 0.1**) and significantly differed between IPF apex and base samples (Wilcoxon test FDR‐corrected *P*‐value < 0.05*) (Figure 2d). Notably, when compared to histological scoring (Figure 1b), Factor1 values were able to better discriminate between IPF apex versus base. Marked differences between IPF apex and base samples were also observed for Factor2 (Wilcoxon test FDR‐corrected *P*‐value < 0.1**) with no distribution overlap (Figure 2e). When considering topographical sampling as a proxy for the different stages of IPF, Factor1 values increased linearly with disease progression (Figure 2f), while a nonlinear U‐shaped relationship was observed for Factor2 (Figure 2g). Altogether, the unbiased integration of multiple omics layers revealed key sources of co‐variation captured by factors associated with fibrosis establishment (Factor1) and progression (Factor2) following distinct linear and nonlinear patterns, respectively.

### Multi‐omics Factor1 top features highlight distinct molecular pathways linked with established fibrosis

**Figure 3:**
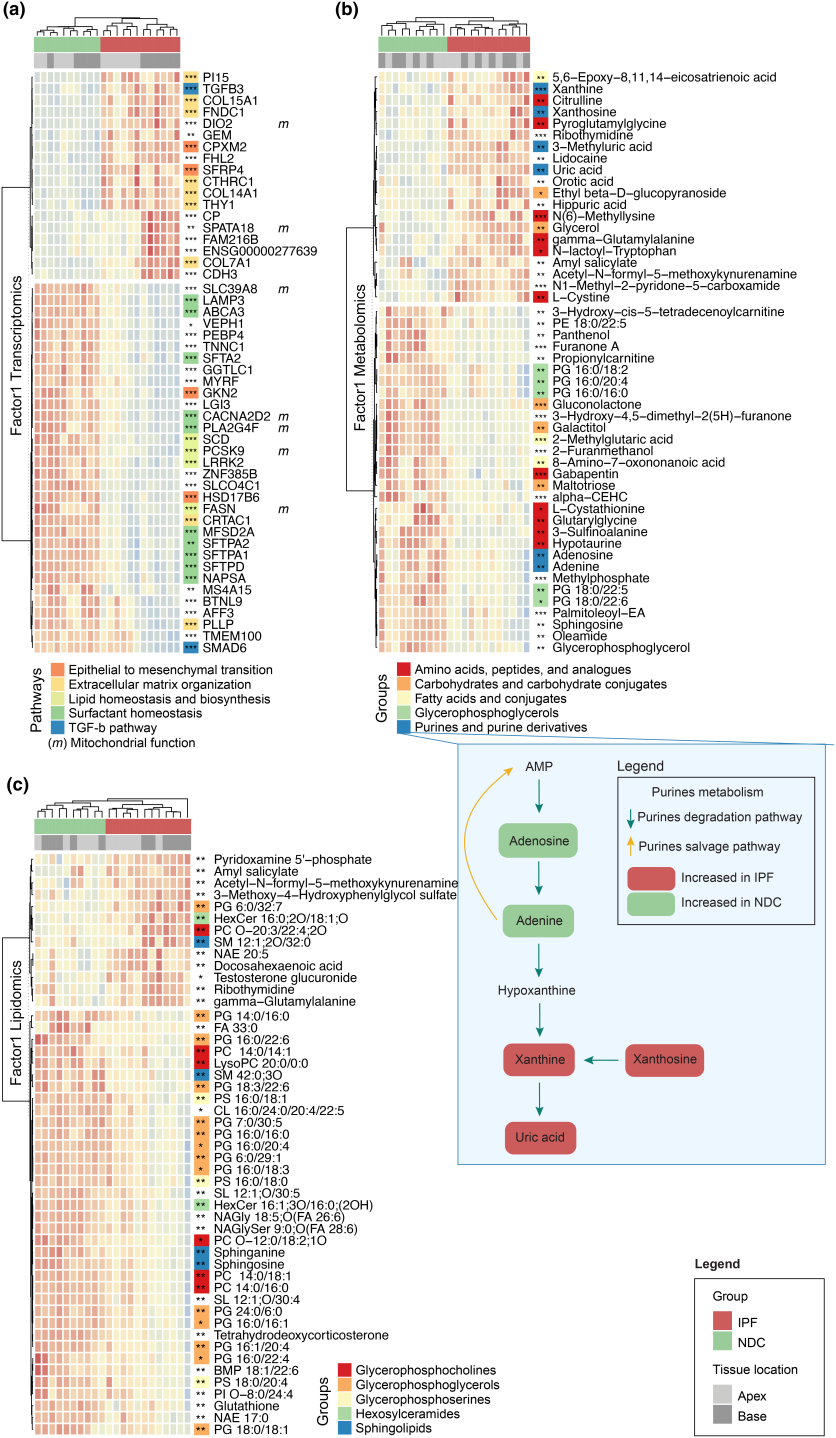
In‐depth characterisation of Factor1. **(a)** Heatmap representing the top 50 genes with the highest loading values for Factor1, associated with disease and age. Each row represents a gene (*z*‐score of normalised gene expression) and each column a sample. **(b)**  Corresponding heatmap for metabolites. Each row represents a metabolite (*z*‐score of normalised metabolites intensities). Purines metabolism pathway (degradation and salvage) with metabolites increased in non‐diseased controls (NDC) (green) or idiopathic pulmonary fibrosis (IPF) (red) samples is highlighted in the orange box. **(c)** Corresponding heatmap for lipids. Each row represents a lipid (*z*‐score of normalised lipids intensities). Column annotations depict disease groups and lung tissue locations. Row annotations refer to literature‐based pathways for transcriptomics and metabolomics, and broad lipid classes for lipidomics. Sample sizes are 6 IPF apex, 6 IPF base, 5 NDC apex and 5 NDC base. Statistics represent the result of FDR‐corrected limma differential gene expression testing for transcriptomics and lm for metabolomics and lipidomics with FDR < 0.05, < 0.01 and < 0.001 represented as *, ** and ***, respectively.

To investigate the molecular aetiology of the first two factors, we used the top 50 MOFA factors loadings of each omics (Supplementary table 2). Maximum distance clustering of MOFA's Factor1 top genes separated samples based on disease status (IPF versus NDC) (Figure 3a). Gene pathways upregulated with disease included extracellular matrix (ECM) synthesis and deposition (*COL7A1*, *COL14A1*, *COL15A1*, *FNDC1*, *PI15*, *CTHRC1* and *THY1*), epithelial–mesenchymal (EMT) transition (*SFRP4*, *CPXM2* and *VEPH1*), and a profibrotic TGFβ isoform (*TGFB3*). Comparatively, gene expression pathways increased in NDC samples were related to surfactant homeostasis (*SFTPA1*, *SFTPA2*, *SFTPD*, *SFTA2*, *ABCA3*, *MFSD2A*, *LAMP3*, *NAPSA*, *PLA2G4F* and *CACNA2D2*), lipid homeostasis and biosynthesis (FASN, PCSK9 and SCD), EMT (*GKN2*) and TGFβ pathway inhibition (*SMAD6*). Interestingly, a subset of genes (noted with ‘m’) characterising the first factor were linked to mitochondrial function (*SLC39A8*, *PLA2G4F*, *CACNA2D2*, *FASN*, *DIO2* and *PCSK9*) and predominantly downregulated in IPF samples. Similar to gene expression, maximum distance clustering of the top metabolites explaining Factor1's variation demonstrated a clear separation of samples based on disease status (Figure 3b). Differences in amino acid or derivatives were observed, either increased (e.g. citrulline, pyroglutamylglycine, N(6)‐Methyllysine, gamma‐Glutamylalanine and N‐lactoyl‐Tryptophan) or decreased (e.g. gabapentin and glutarylglycine). Specifically, increased levels of glutathione metabolism intermediates (gamma‐glutamylalanine, pyroglutamylglycine and L‐cystine) and decreased levels of precursors (L‐cystathionine and glutarylglycine) and glutathione itself were observed in IPF tissues. The hypotaurine/taurine biosynthesis pathway, which partially overlaps with GSH metabolism, showed increased levels of several members in NDC samples (L‐cystathionine, 3‐sulfinoalanine and hypotaurine), except for L‐Cystine, which was increased in IPF lung explants. Purine pathway members were among the top features for Factor1 (Figure 3b blue box). Adenine and adenosine were increased in NDC samples, while downstream molecules (xanthine, xanthosine and uric acid) were increased in IPF tissues. Finally, investigation of the top lipidomics features characterising Factor1 revealed a similar trend as the metabolomics dataset; a clear separation between IPF and NDC samples (Figure 3c). The majority of Factor1's top lipids were found to be increased in NDC samples, with 14 of them belonging to the phosphatidylglycerol (PG) family, the second most abundant surfactant phospholipid.^18^, ^19^ Other increased lipids in NDC samples included glycerophosphocholines, glycerophosphoserines and sphingolipids. In contrast, two hexosylceramides lipids family members were increased in IPF tissues. The antioxidant glutathione was increased in NDC explants. Altogether, investigation of the top features of Factor1 revealed well‐established fibrosis pathways (ECM, EMT and TGFβ), in some cases shared across multiple omics layers (e.g. surfactant in both the transcriptomics and lipidomics layers).

### Multi‐omics Factor2 top features reveal molecular pathways specific to disease progression

**Figure 4:**
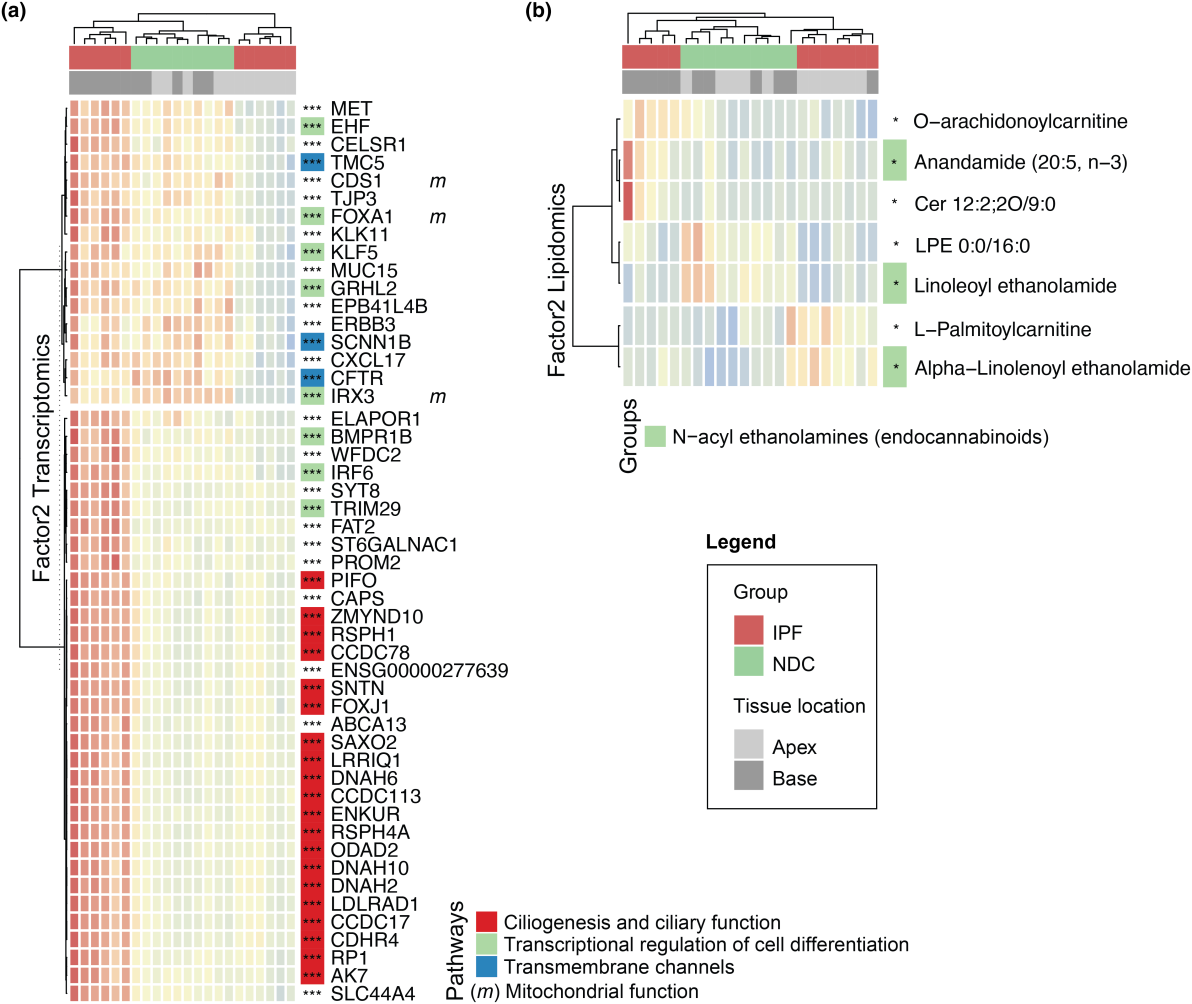
In‐depth characterisation of Factor2. **(a)** Heatmap representing the top 50 genes with the highest loading values for Factor2, associated with disease progression. Each row represents a gene (*z*‐score of normalised gene expression) and each column a sample. **(b)** Corresponding heatmap for lipids. Each row represents a lipid (*z*‐score of normalised lipids intensities). Column annotations depict disease groups and lung tissue locations. Row annotations refer to literature‐based pathways for transcriptomics and broad lipid classes for lipidomics. Sample sizes are 6 IPF apex, 6 IPF base, 5 NDC apex and 5 NDC base. Statistics represent the result of FDR‐corrected limma differential gene expression testing for transcriptomics and lm for metabolomics and lipidomics with FDR < 0.05, < 0.01 and < 0.001 represented as *, ** and ***, respectively.

Investigation of the top features explaining Factor2 revealed pathways differentiating IPF apex (pre‐fibrosis) from base (fibrosis) samples. Two gene expression patterns were identified: (1) increased expression only in IPF base samples (Figure 4a bottom) and (2) increased expression in IPF base but decreased in IPF apex samples with intermediate levels in NDC samples (Figure 4a top). The second pattern explains the nonlinear pattern observed in Factor2. The first gene expression pattern found in IPF base samples was associated with ciliogenesis, including *FOXJ1* and genes involved in cilia structure (DNAH genes), motility (*SAXO2* and *SNTN*) and mucociliary clearance (*ODA2*, *LDLRAD1*, *RSPH4A*, among others). In contrast, the second pattern showed reduced expression of genes involved in cell differentiation regulation (*TRIM29*, *IRF6*, *EHF*, *IRX3*, among others) and transmembrane channel genes (*TMC5*, *CFTR* and *SCNN1B*) in pre‐fibrotic IPF apex samples. Many of these genes play a role in embryogenesis (*FOXA1*, *MET*, *CELSR1*, *IRX3*, *KLF5*, *GRHL2* and *ERBB3*) and mitochondrial function (*FOXA1*, *CDS1* and *IRX3*). Top lipidomics features for Factor2 also revealed IPF progression‐specific features, separating IPF bases and apices (Figure 4b). IPF bases showed elevated levels of long‐chain acylcarnitines and O‐arachidonoylcarnitine, and IPF apices had higher L‐Palmitoylcarnitine, with NDC samples exhibiting low levels of these compounds. A notable pattern linked to endocannabinoid signalling was observed, varying with the disease progression status. Anandamide, which transforms into pro‐inflammatory arachidonic acid via fatty acid amide hydrolase (FAAH), was increased in IPF bases. Conversely, linoleoylethanolamide (LEA), a natural FAAH inhibitor^20^ reducing arachidonic acid production, was elevated in NDC tissues but low in IPF samples. Of note, a‐Linoleoylethanolamide (a‐LEA) levels were increased in IPF apices. In summary, fibrosis progression was associated with ciliogenesis, transcriptional regulation of cell differentiation and discrete changes in lipid profiles.

### Machine learning algorithms predict fibrotic and pre‐fibrotic signatures in independent datasets

**Figure 5:**
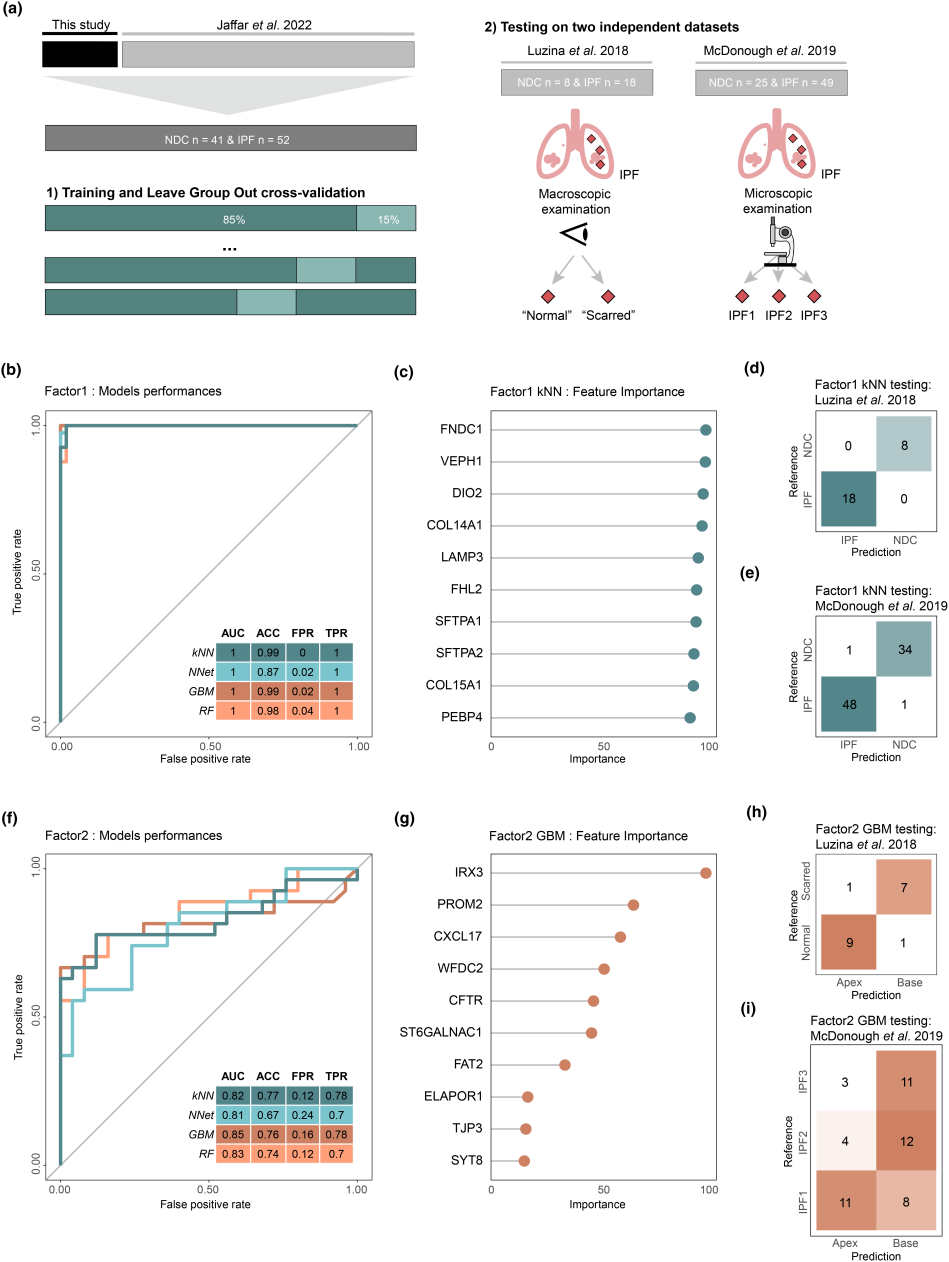
Machine learning and external validation using the transcriptomics data modality. **(a)** Workflow for machine learning models training and testing strategy using the current dataset with previously published data using a leave‐one‐group‐out cross‐validation (LOGCV) approach (left) followed by testing of the best‐performing model on two independent testing datasets (right). The four machine learning algorithms used for both Factor1 and Factor2 were k‐nearest neighbours (kNN), neural network (NNet), stochastic gradient boosting (GBM) and random forest (RF). The following metrics were used to assess each model's prediction capability: area under the receiver operating characteristic (ROC) curve (AUC), accuracy (ACC), false‐positive rate (FPR) and true‐positive rate (TPR). **(b)** ROC curves depicting the FPR against the TPR for the four different algorithms in discriminating disease status (idiopathic pulmonary fibrosis (IPF) versus non‐diseased controls (NDC)) using the top 50 Factor1 genes as input. **(c)** Scaled features importance (genes) for the kNN classification model for Factor1. **(d, e)** Confusion matrices for the independent testing datasets using the kNN classification model for Factor1. **(f)** ROC curves for the four different machine learning algorithms for predicting IPF disease progression (IPF base versus IPF apex) using the top 50 Factor2 genes as input. **(g)** Scaled features importance (genes) for the stochastic gradient boosting classification model for Factor2. **(h, i)** Confusion matrices for the independent testing datasets using the stochastic gradient boosting classification model for Factor2. Sample sizes are **(b)** 41 NDC, 52 IPF; **(f)** 26 base, 26 apex; **(d)** 8 NDC, 18 IPF; **(e)** 35 NDC, 49 IPF; **(h)** 8 scarred IPF, 10 normal IPF; **(i)** 19 IPF1, 16 IPF2, 14 IPF3.

Given the importance of the transcriptomics layer in MOFA‐discovered Factor1 and Factor2, we evaluated the predictive performance of their top genes using machine learning (ML). To ensure accurate ML model training and performance estimation, the presented transcriptomics dataset was integrated with a larger published dataset from the same patient cohort.^21^ Four ML models were trained and compared using a leave‐one‐group‐out cross‐validation (LOGCV) approach. To validate the best‐performing ML models, two publicly available transcriptomics datasets from GEO archives that utilised differential site sampling to investigate IPF were reprocessed.^15^, ^16^ These included macroscopic sampling of visually appearing scarred and normal regions of tissue^15^ and histological classification of samples from the apex, middle and base of explanted IPF lungs into stages of IPF progression^16^ (IPF1 = early‐stage fibrosis, IPF2 = mid‐stage fibrosis, IPF3 = end‐stage fibrosis) alongside NDC samples (Figure 5a). The disease classification performance (Factor1) was first evaluated. All the ML models showed strong disease‐discriminating performance properties, achieving an Area Under the Curve AUC of 1 for all tested models (Figure 5b). The k‐nearest neighbours model had the highest mean accuracy (ACC: 0.99), the lowest false‐positive rate (FPR: 0) and a true‐positive rate of 1 (TPR: 1). Feature importance evaluation of the latter revealed genes with high discriminating power, including fibronectin gene *FNDC1*, followed by *VEPH1*, collagen genes *COL14A1* and *COL15A1*, surfactant protein genes *SFTPA1* and *SFTPA2*, among others (Figure 5c). The same model was then used to compare disease prediction in two independent datasets, generating a perfect confusion matrix observed for the first dataset (Figure 5d) and highly discerning classification for the other (Figure 5e). The prediction of disease progression using Factor2 top genes was evaluated next, categorising IPF samples into two classes based on the degree of fibrosis: early fibrosis (IPF apex) and late‐stage fibrosis (IPF base). The stochastic gradient boosting ML model performed marginally better than other ML models with an AUC of 0.85, ACC of 0.76, FPR of 0.16 and TPR of 0.78 (Figure 5f). Feature importance evaluation revealed the transcription factor *IRX3* to be the most important for distinguishing between base and apex samples. Other strong predictors among Factor2 included several genes involved in epithelial functions (*PROM2*, *WFDC2*, *CFTR*, among others) (Figure 5g). External dataset validation showed concordant classification of Luzina *et al*. samples, indicating similarity between their ‘normal’ samples and the current study's apex samples (Figure 5h). McDonough *et al*. samples exhibited more variability (Figure 5i), with ‘mid’ and ‘late‐stage’ classifications aligning with IPF base prediction, while some early‐stage (IPF1) samples were split between base and apex predictions, potentially indicating early cellular dysfunction. Overall, validation of the MOFA‐identified genes on external datasets demonstrated their generalisability beyond the initial discovery dataset.

## Discussion

Using an unbiased multi‐omics approach, we aimed to model the leading edge of fibrosis to identify novel therapeutic targets. MOFA identified five sources of co‐variation, revealing molecular signals that might have been missed with single omic analysis. Factor1 represented variability that distinguished IPF from NDC tissues, while gradually and linearly increasing with disease stage in addition to correlating with histological scoring and age. The most important findings from the current study, however, revolve around Factor2, which discriminated between early (IPF apex) and later (IPF base) stages of IPF, following an unconventional nonlinear dynamic. Unlike Factor1, Factor2 was not associated with age and revealed pathophysiological events which may be driving a pre‐fibrotic ‘at risk’ microenvironment towards pathological tissue remodelling.

In‐depth characterisation of the molecular aetiology of Factor1 revealed a downregulation of surfactant signals observed in IPF in both the cellular transcripts and lipids compartments. This included key genes associated with surfactant biogenesis, transport and homeostasis—orchestrated by AEC2s—as well as lipids of the PG family. Indeed, PG lipids are the second most abundant lipid class in adult rodent surfactant^22^ and have been demonstrated to exhibit spatial localisation specifically within the airway epithelium.^23^ As expected, TGFβ transcript levels were increased in IPF samples while the expression of its negative regulator SMAD6^24^ was decreased. This goes in line with the increased expression of genes related to EMT (*SFRP4* ^25^ *and CPXM2* ^26^), a process that contributes to fibrosis, in IPF explants compared with NDC. We also observed an upregulation of collagen and other matrix‐associated genes (*CTHRC1* ^27^ and *FNDC1* ^28^ among others) previously identified as potential biomarkers of IPF.^27^, ^29^, ^30^, ^31^ Notably, *FNDC1* was the most important feature predicting established disease in our ML model for Factor1. Further investigation of genes associated with surfactant and lipid homeostasis (*SPATA18*, *SLC39A8*, *PLA2G4F*, *CACNA2D2*, *FASN* and *PCSK9*) also revealed their implication in mitochondrial function. While the expression of these genes may be reflecting differences in cell composition (e.g. loss of AEC2s in IPF), their association with mitochondrial dysfunction was further supported by observed alterations in the metabolomics modality. Indeed, oxidative stress and tissue hypoxia have been reported to induce the degradation of adenosine,^32^, ^33^ which results in the release of certain by‐products, including hypoxanthine, xanthine and uric acid.^34^ Low adenosine and high hypoxanthine levels have been reported in IPF lungs,^35^ and uric acid was identified as an important signal that triggers inflammation and fibrosis in mice.^36^ Other affected metabolic pathways in IPF explants included a decreased hypotaurine/taurine metabolism signal, in line with previous studies demonstrating the protective role of hypotaurine/taurine metabolism against oxidant‐induced lung injury and fibrosis.^37^, ^38^ The decrease in GSH pathway precursors indicates a redox imbalance, suggesting vulnerability to metabolic inflammation and lung injury not only in established fibrosis (IPF base), as previously reported,^39^ but also in the pre‐fibrotic IPF apices. Both gene expression and metabolic signals should be interpreted in consideration of the confounding effect related to a shift in cell composition, such as well‐described alterations in AEC2 and fibroblast proportions in IPF.^40^ In summary, Factor1 revealed a multi‐omics signature associated with AEC2 dysfunction, tissue remodelling, and oxidative stress in both fibrosed bases and pre‐disease apices, indicating early dysfunction of the epithelial compartment in IPF. Given that the biological entities encapsulated within Factor1 demonstrate alterations during the early stages of the disease, they hold promise as potential biomarkers for early disease detection. Particularly, metabolites identified by Factor1 emerge as strong biomarker candidates as a result of their stability and ease of measurement.

Juxtaposed with the linear and gradual cellular events leading to fibrosis underlined by the first factor, we discovered a unique molecular signature linked to disease progression, characterised by a nonlinear dynamic (Factor2) predominantly explained by the transcriptomics layer. Ciliogenesis and cilia function signals were specifically increased in the latest stage of IPF. Primary cilia are not usually found in healthy adult alveoli, but they may briefly reappear during lung injury, possibly indicating their role in wound healing.^41^, ^42^ High cilia‐related gene expression in IPF lungs has been linked to increased honeycombing and activation of the SHH pathway,^43^ which is known to be important in the lung during embryonic development.^44^ Factor2 also highlighted an intriguing pattern of genes characterised by an intermediary expression in healthy tissue (NDC samples), a downregulation in pre‐fibrosis (IPF apex) and an upregulation in established fibrosis (IPF base). Many of these genes are involved in transcriptional regulation of different processes, including EMT (*FOXA1*, *GRHL2 and ERBB3*)^45^, ^46^, ^47^ or carcinogenesis (*EHF*, *TRIM29*, *KLF5* and *MET*),^48^, ^49^, ^50^, ^51^ and also play a crucial role in embryonic lung development (*FOXA1*, *CELSR1*, *IRX3*, *KLF5* and *GRHL2*).^52^, ^53^, ^54^, ^55^, ^56^ Of particular interest is the transcription factor *IRX3*, the top most important feature of the best‐performing ML model for Factor2, known not only for its roles in energy homeostasis^57^ and mitochondrial function,^58^ but also for orchestrating an array of cellular processes crucial for developmental biology, such as proximo‐distal morphogenesis of the developing lung^54^ or lung angiogenesis.^59^ Factor2 top lipidomics features identified elevated long‐chain acylcarnitines in IPF samples and in particular L‐Palmitoylcarnitine in IPF apices, reflective of mitochondrial dysfunction. Indeed, acylcarnitines are produced during carnitine metabolism for FA oxidation in the mitochondria. Notably, their accumulation in serum and tissues has been associated with apoptosis via glutathione depletion and oxidative stress induced by mitochondrial dysfunction.^60^, ^61^, ^62^, ^63^ This underlines the significance of the observed increase in L‐Palmitoylcarnitine in IPF apices as a potential biomarker for mitochondrial dysfunction. Changes in lipids part of the endocannabinoid system, such as high anandamide levels in IPF base samples, were observed along Factor2. Studies have associated elevated anandamide levels with chronic tissue injury,^64^, ^65^ the progression of schistosoma‐induced liver fibrosis,^66^ and pulmonary fibrosis progression in patients and a bleomycin mouse model of IPF^67^ while modulating the endocannabinoid signalling showed potential in reducing collagen production and myofibroblast activation.^68^ Interestingly, increased anandamide levels were only evident in IPF bases, while higher levels of LEA, which can attenuate anandamine hydrolysis to arachidonic acid,^20^ were observed in NDC samples. Altogether, Factor2 demonstrated an altered lipidomic profile that aligned with our finding of decreased expression of mitochondria‐related genes, such as *IRX3*, specifically in pre‐fibrotic apices. For example, increased mitochondrial fragmentation and decreased mitochondrial complex IV activity have been observed with *in vitro* inhibition of *IRX3*.^69^ We propose here a causal link between reduced *IRX3*, mitochondrial dysfunction and lipid metabolism inflammation that can potentially account for the early tissue injury and fibrosis spreading in IPF apices. This pattern could be indicative of dynamic shifts in cellular processes during different stages of IPF progression. One hypothesis is that the intermediary expression in healthy tissue represents the equilibrium in lung epithelial function, while downregulation in pre‐fibrosis might signify the early stages of injury and dysregulation, leading to the subsequent aberrant tissue repair response and upregulation in established fibrosis. Eventually, the system reaches a ‘tipping point’ where these become aberrantly reactivated (IPF base). This aligns with the idea proposed by Froidure *et al*.^4^ that such pathways may follow a nonlinear pattern governed by chaos. This type of dynamic is sensitive to initial conditions, and small changes can have large irreversible effects, such as the tissue remodelling seen in late‐stage IPF.

While this study offers valuable insights into IPF progression, it is crucial to recognise its limitations, including the small sample size and potential variability in cellular composition across lung explant sampling sites. Nevertheless, our findings revealed strikingly clear and distinct molecular signatures between groups, underscoring robust and conserved mechanisms associated with disease progression. Additionally, we employed a machine learning approach to validate the reliability and generalisability of our gene expression findings. This included testing our predictive models on the only two other studies to our knowledge with comparable sample types.^15^, ^16^ Indeed, our validation of Factor1 genes revealed a consistent signature that discriminated between NDC and IPF samples regardless of sample site or histopathological severity. In contrast, Factor2 genes displayed strong training performance which performed well in one of two datasets in validation. We hypothesise that the Factor2 signature may be particularly sensitive to sample handling and processing given the contrasting study‐specific performance. Future work may allow careful dissemination of how and why these genes are initially downregulated in pre‐fibrosed areas and then reactivated in extensively fibrosed regions.

Future investigations may focus on examining the cellular heterogeneity present in IPF lung explants from different disease stages, for example through the utilisation of single‐cell sequencing. Notably, recent pioneering studies have laid the foundation for the IPF single‐cell atlas,^70^ paving the way for future investigations to delve into the cellular heterogeneity across various disease stages. Moreover, the integration of other emerging technologies, such as single‐cell metabolomics,^71^ could offer valuable insights into the diverse molecular profiles and metabolic dynamics within specific cell populations. In summary, we identified sources of multi‐omics co‐variations linked with IPF establishment and progression. We postulate that the multi‐omics signature in IPF apices that was revealed by Factor2 represents the ‘leading edge of fibrosis’, an inflection point that marks a turning point in IPF progression.

## Methods

### Study population/ethics and sampling

The study received ethical approval from the Alfred Human Ethics Committee (#468/14) and the Australian Red Cross Blood Service Ethics Committee, meeting the Australian National Statement on Ethical Conduct in Human Research (2007). Tissue samples were obtained from the Alfred Lung Fibrosis Biobank, and written informed consent was obtained from all participants or their families. Lung tissue was obtained from patients with end‐stage interstitial lung disease or from diseased organ donors whose lungs were declined for transplantation.

### Histological scoring

Lung tissues were fixed in 4% paraformaldehyde and embedded in paraffin blocks. Sections of 4 μm were cut for Masson's trichrome staining. Blinded scoring of the lung histopathology was performed according to the Ashcroft scoring methodology.^72^

### Transcriptomics data acquisition and processing

Total RNA was extracted from lung tissues as described in Jaffar *et al.* ^21^ Raw fastq files were processed with RNASik v1.5.0,^73^ aligned using STAR v2.5.2b against the GRCh38.104 reference genome and read counts were quantified to annotated genes using featureCounts.^74^ Annotated protein‐coding genes were retained and low expression genes filtered using the filterByExpr function from edgeR v3.36.0 before normalisation with vsn R package v3.62.0. For integrated analysis with previous datasets, raw fastq files were downloaded from the SRA from Jaffar *et al*. (GSE213001),^21^ Luzina *et al*. (GSE99621)^15^ and McDonough *et al*. (GSE124685).^16^ Files were processed using the above pipeline, including batch effect removal using ComBat‐seq^75^ with study as the batch effect and disease (NDC or IPF) as a biological condition.

### LCMS sample processing and data acquisition

Frozen lung tissues were crushed with a stainless steel multisample pulveriser dipped in liquid nitrogen. The resulting powder was extracted using 80% MeOH with 1 μM CHAPS, CAPS and PIPES internal standards for metabolomics and CHCl3:MeOH:H_2_O (1:3:1, v/v) spiked with 45 nM Cer(35:1) ceramide and 25 nM SM(35:1) and 10 μM BHT for lipidomics. After vortexing, sonication and centrifugation, the supernatant was transferred to Eppendorf tubes, and a pooled QC was prepared with blank samples for each analysis. Lipidomics samples were evaporated and solubilised in MeOH:BuOH:H_2_O (4.5:4.5:1, v/v) mixture, vortexed, sonicated and centrifuged. LCMS data were acquired on a Q‐Exactive Orbitrap mass spectrometer (Thermo Fisher) coupled with the Dionex Ultimate 3000 RS (Thermo Fisher) high‐performance liquid chromatography system in a single batch and randomised to account for system drift.

### LCMS data analysis

LCMS data were processed using the metabolome‐lipidome‐MSDIAL pipeline (see data and materials availability section). MS‐DIAL v4.7 was used for peak detection and alignment against the MassBank v2021.02 and MS‐DIAL internal lipid databases with the following parameters: minimum peak amplitude of 100 000, retention time tolerance parameter of 1 min and mass tolerance of 0.002 Da with gap‐filling. Peak intensities were normalised using probabilistic quotient normalisation (PQN), followed by random forest missing data imputation and subsequent generalised logarithmic (glog) transformation using the pmp R package v1.6.0. Filtered peaks were mapped to the Human Metabolome database (HMDB) v4.202107 with a mass tolerance of 0.002 Da and annotated features were curated using a combination of the MS‐DIAL fill percentage and signal‐to‐noise ratio values, along with visual confirmation.

### Multi‐omics data integration

Normalised transcriptomics, metabolomics and lipidomics matrices were used as an input for Multi‐Omics Factor Analysis 2 (MOFA+) v1.4.0. This unsupervised Bayesian approach infers a low‐dimensional representation of the data (latent factors) capturing overall sources of variability. Default parameters were used. Characterisation of the MOFA2 model included the quantification of the variance explained (*R* ^2^) by each factor in each view as well as the assessment of the top 50 weights in each omics modality, which can be interpreted as a measure of feature importance. Top feature characterisation into pathways was performed using a literature‐based search (genes) or grouping into broad molecular categories (lipids and metabolites).

### Machine learning

The top 50 genes with the highest weights in Factor1 and Factor2 were used as predictor variables for IPF and NDC, and early (IPF apex) and late‐stage disease (IPF base) respectively. To increase the sample size for model training and performance evaluation, Jaffar *et al*.'s previously published dataset^21^ was included. Validation and performance estimations of four models were performed using a leave‐group‐out cross‐validation approach using the caret R package, in which the data were folded 50× into 85% training data and 15% validation data. The best‐performing models, as determined using evalm from MLeval v0.3 R package, were selected based on the maximum mean area under the ROC curve and accuracy values. Feature importance was estimated using the varImp function. The models were tested on two additional datasets,^15^, ^16^ and a confusion matrix was generated for each dataset and factor.

### Statistical analysis and data visualisation

Downstream analyses and figures were generated using R statistical software v4.1.1. Differences between groups were addressed using non‐parametric Wilcoxon rank‐sum testing with FDR correction for multiple testing, and PCA was performed on normalised transcriptomics, metabolomics and lipidomics matrices using the prcomp function. Transcriptomics differential expression analysis was performed using the R package limma v3.56.2 to identify differentially expressed genes (FDR adjusted *P*‐values < 0.05) to compare disease groups, apex/base sampling and disease progression. For the analysis of differentially abundant metabolites and lipids, linear modelling (lm function in stats v3.4.1) was used for each feature followed by FDR adjustment of *P*‐values. Pearson correlations between factors and covariates were calculated using the correlate_factors_with_covariates function in the MOFA v1.4.0 R package (significant correlations with *P*‐value < 0.05 are presented). Data visualisation was performed using ggplot2 R package v3.3.5 and heatmaps drawn with ComplexHeatmap v2.10.0 R package with clustering method complete for rows and Maximum for columns. A fixed random‐number seed value of 2 was set for reproducibility.

## Author contributions

**Céline Pattaroni:** Formal analysis; visualization; writing – original draft. **Christina Begka:** Data curation; formal analysis; writing – original draft. **Bailey Cardwell:** Formal analysis; visualization; writing – original draft. **Jade Jaffar:** Investigation; methodology. **Matthew Macowan:** Methodology. **Nicola L Harris:** Writing – review and editing. **Glen P Westall:** Conceptualization; funding acquisition; writing – review and editing. **Benjamin J Marsland:** Conceptualization; funding acquisition; writing – review and editing.

## Conflict of interest

The authors declare no conflict of interest.

## Supporting information


Supplementary figure 1
Supplementary figure 2
Supplementary table 1
Supplementary table 2

## Data Availability

Raw transcriptomics data for this study are freely available as NCBI GEO Dataset GSE213001. Pipeline for LCMS data processing are available at https://github.com/respiratory‐immunology‐lab/metabolome‐lipidome‐MSDIAL. Annotated normalised metabolomics and lipidomics intensity tables are available as Mendeley datasets (doi: 10.17632/4zr8jjmzhy.1).
