# Supplementary material for: Multi‐omics integration reveals a nonlinear signature that precedes progression of lung fibrosis
Source: Clin Transl Immunology. 2024 Jan 24;13(1):e1485. doi: 10.1002/cti2.1485 (PMC10807351; doi:10.1002/cti2.1485)
Supplement: Supplementary file 1 — Supplementary figure 1 Supplementary figure 2 Supplementary table 1 Supplementary table 2 [file CTI2-13-e1485-s001.pdf]

SUPPLEMENTARY INFORMATION

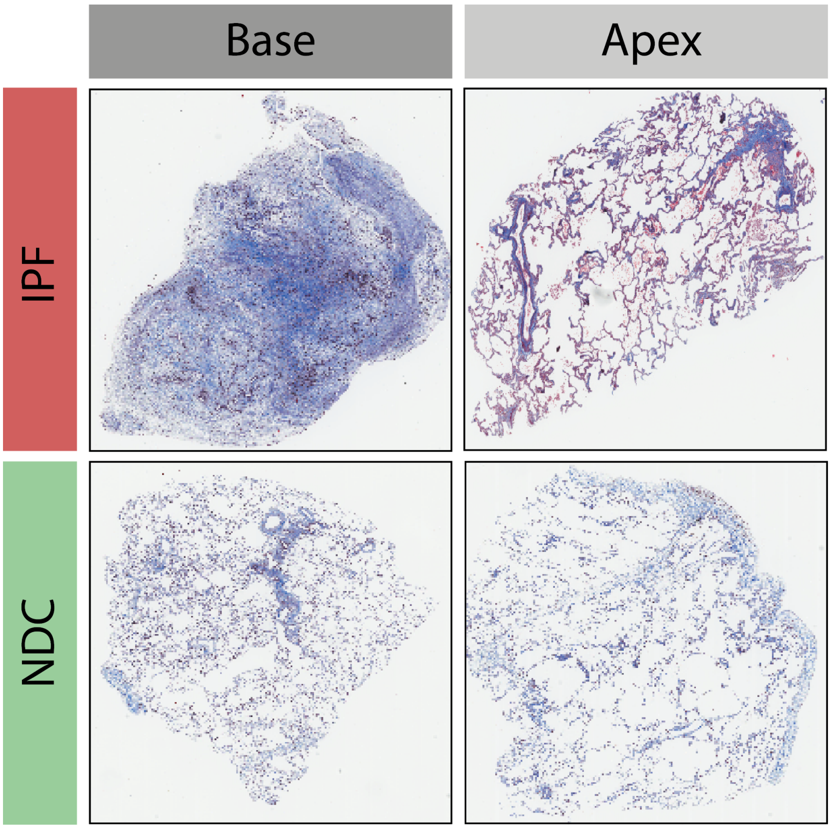

Supplementary figure 1. Lung explants histology results. Representative lung sections of IPF base, apex and NDC base, and apex samples with Masson's trichrome staining.

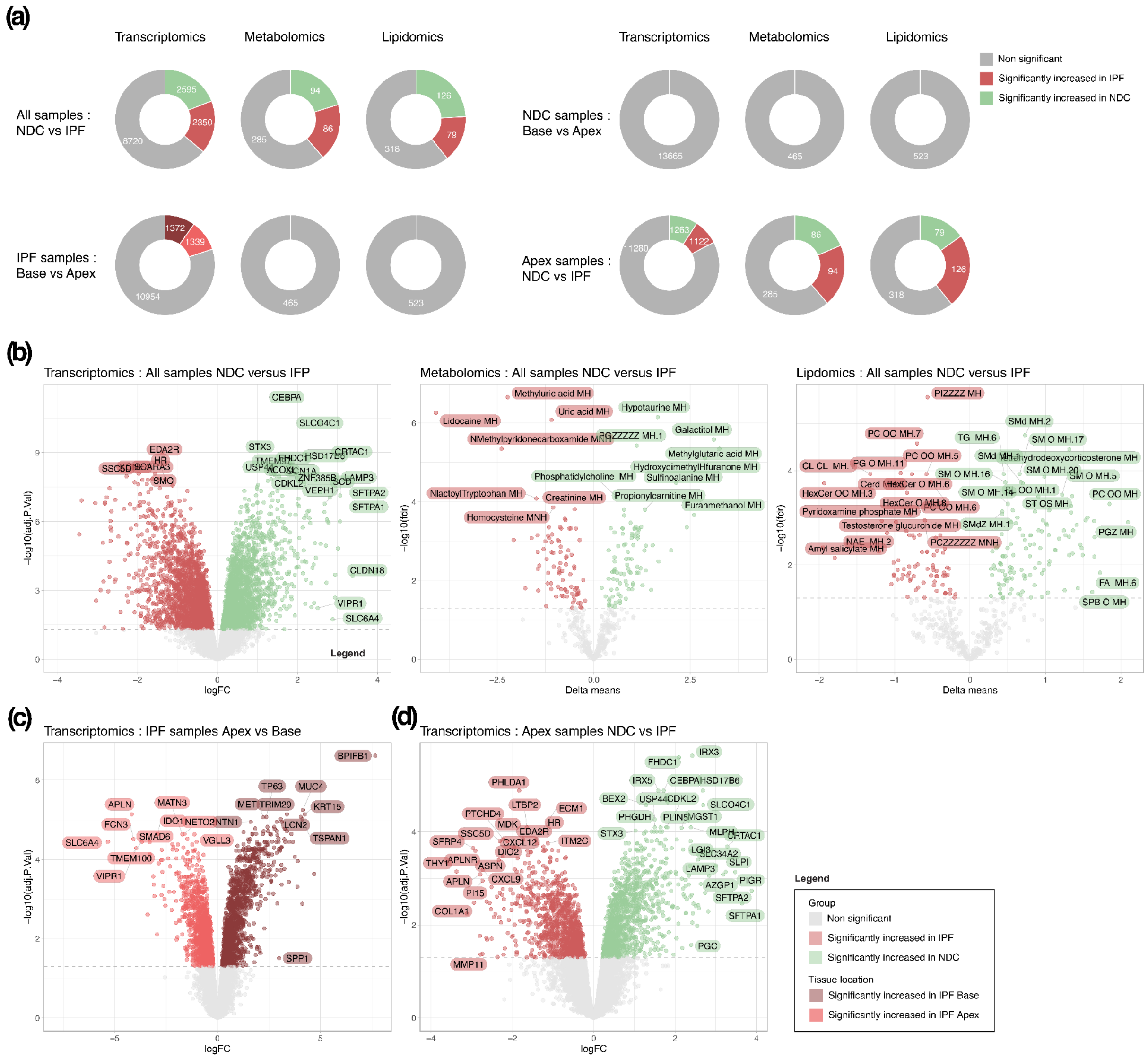

Supplementary figure 2. Standard differential expression/abundance testing. **(a)** Number of DE expressed / DA features for each omics with each comparison using limma or linear modelling **(b)** Volcano plots depicting differential expression/abundance testing results for disease (IPF versus NDC) testing for each individual omics **(c)** Volcano plot depicting the differential expression testing results for IPF samples base versus apex comparisons **(d)** Volcano plot depicting the differential expression testing results for apex samples NDC versus IPF comparisons.

Supplementary Table 1. Patients characteristics and samples metadata.

Sample ID	Group	Sample type	Gender	Age	BMI	Smoking	Ashcroft score	Years since IPF onset	Treatments
19	IPF	Apex	F	56	20.4	Never	1.9	4	Nil
19	IPF	Base	F	56	20.4	Never	7.6	4	Nil
23	IPF	Apex	M	61	24.4	Never	6.4	1	Pirfenidone
23	IPF	Base	M	61	24.4	Never	7.5	1	Pirfenidone
28	IPF	Apex	F	59	28.6	Ex	3.7	2	Prednisone
28	IPF	Base	F	59	28.6	Ex	6.9	2	Prednisone
39	IPF	Apex	M	67	28.1	Never	7.1	8	Pirfenidone
39	IPF	Base	M	67	28.1	Never	7.9	8	Pirfenidone
43	IPF	Apex	F	70	28.3	Ex	8.0	5	Nil
43	IPF	Base	F	70	28.3	Ex	8.0	5	Nil
45	IPF	Apex	F	64	25.2	Never	4.7	5	Pirfenidone
45	IPF	Base	F	64	25.2	Never	7.3	5	Pirfenidone
24	NDC	Apex	F	44			0.2		
24	NDC	Base	F	44			0.4		
26	NDC	Apex	M	28		Ex	0.4		
26	NDC	Base	M	28		Ex	0.6		
30	NDC	Apex	NA	35			0.5		
30	NDC	Base	NA	35			0.3		
34	NDC	Apex	F	67			0.1		
34	NDC	Base	F	67			0.1		
50	NDC	Apex	M	42			0.1		
50	NDC	Base	M	42			1.4		

Supplementary Table 2. MOFA top loadings results for Factor1 and Factor2 with corresponding differential expression/abundance testing FDR-corrected *P*-values.

**Factor 1**								
Transcriptomics	Value	FDR	Metabolomics	Value	FDR	Lipidomics	Value	FDR
COL15A1	1.0	1.6E-07	NMethylpyridonecarboxamide MNH	1.0	4.5E-06	HexCer OO MH.3	1.0	0.00
LGI3	1.0	9.0E-08	Galactitol MH	0.8	2.6E-06	PGZ MH	1.0	0.00
COL7A1	1.0	1.8E-06	Lidocaine MH	0.8	5.5E-07	PC OO MH	0.9	0.00
CRTAC1	1.0	9.4E-10	Methyluric acid MH	0.8	2.2E-07	PI O MH	0.9	0.00
DIO2	1.0	1.5E-07	Methylglutaric acid MH	0.8	4.5E-06	PG MH	0.9	0.00
ABCA3	1.0	1.0E-06	Sulfinoalanine MH	0.8	3.3E-05	Sphingosine MH	0.9	0.00
LAMP3	1.0	7.4E-08	Furanmethanol MH	0.8	2.2E-04	PGZZZ MH	0.9	0.00
SLC39A8	1.0	3.9E-07	HydroxydimethylHfuranone MH	0.8	7.8E-06	PGZZZZZ MH	0.9	0.00
CACNA2D2	0.9	8.1E-07	Hypotaurine MH	0.7	7.0E-07	PGZZZZ MH	0.9	0.00
VEPH1	0.9	5.2E-08	NlactoylTryptophan MH	0.7	8.1E-05	PGZ MH.1	0.8	0.01
PLA2G4F	0.9	1.5E-05	Methylphosphate MH	0.7	1.3E-03	NAGly OFA MH.3	0.8	0.00
MYRF	0.9	3.8E-05	Glycerol MH	0.6	8.3E-04	HexCer OOH MH.1	0.8	0.00
FNDC1	0.9	1.1E-07	Glutarylglycine MH	0.6	5.5E-03	CL CL MH.1	0.8	0.00
PEBP4	0.9	9.9E-07	AcetylNformylmethoxykynurenamine MNa	0.6	1.9E-03	Sphinganine MH	0.8	0.00
ZNF385B	0.9	1.6E-08	Sphingosine MH	0.6	7.3E-04	Tetrahydrodeoxycorticosterone MH	0.8	0.00
HSD17B6	0.9	1.5E-09	Hippuric acid MNaH	0.6	9.2E-04	PG PG MH.1	0.8	0.01
SCD	0.8	1.7E-07	Palmitoleoylethanolamde MH	0.6	6.4E-03	PG MH.1	0.8	0.00
COL14A1	0.8	3.6E-08	PGZZ MH	0.6	4.9E-03	SL O MH.1	0.8	0.00
SFRP4	0.8	9.0E-08	Propionylcarnitine MH	0.6	1.6E-04	NAE MH.1	0.8	0.00
GGTLC1	0.8	7.3E-07	Gluconolactone MH	0.6	4.4E-04	LysoPC MH.3	0.8	0.00
MS4A15	0.8	1.2E-06	Ethyl betaDglucopyranoside MNaH	0.6	5.0E-03	SL O MH	0.8	0.01
PI15	0.8	3.6E-07	LCystathionine MH	0.6	1.7E-03	Amyl salicylate MH	0.8	0.00
SPATA18	0.8	8.1E-04	Xanthine MH	0.6	8.3E-04	SM O MH.5	0.7	0.00
SLCO4C1	0.8	5.1E-11	PGZZZZZ MH.1	0.5	3.7E-06	PC MH	0.7	0.00
BTNL9	0.8	4.9E-04	LCystine MH	0.5	7.1E-04	PG MH.2	0.7	0.01
CPXM2	0.8	2.8E-07	PGZZZZZZ MH	0.5	4.3E-04	PSZ MH	0.7	0.00
CP	0.8	1.7E-03	Xanthosine MH	0.5	2.8E-03	AcetylNformylmethoxykynurenamine MH	0.7	0.01
AFF3	0.8	3.0E-05	Adenine MH	0.5	6.4E-03	Pyridoxamine phosphate MH	0.7	0.00

SFTPD	0.8	1.1E-05	Glycerophosphoglycerol MH	0.5	4.3E-04	PCZ MH.1	0.7	0.00
SMAD6	0.8	3.8E-03	alphaCEHC MNaH	0.5	1.7E-03	PSZZZZ MNH	0.7	0.01
LRRK2	0.8	1.9E-06	Aminooxononanoic acid MH	0.5	4.7E-04	BMP BMP MNH	0.6	0.01
SFTA2	0.8	8.7E-06	gammaGlutamylalanine MH	0.5	1.6E-03	NAGlySer OFA MH.3	0.6	0.00
TMEM100	0.8	5.3E-03	Oleamide MH	0.5	6.4E-04	PS MH	0.6	0.02
THY1	0.7	1.2E-07	Furanone A MH	0.5	6.1E-04	PGZZZZ MH.1	0.6	0.00
MFSD2A	0.7	3.7E-07	Maltotriose MH	0.5	3.2E-03	gammaGlutamylalanine MH	0.6	0.01
SFTPA1	0.7	2.5E-07	Gabapentin MH	0.5	5.1E-04	PG MH.3	0.6	0.04
PLLP	0.7	1.8E-04	PEZZZZZ MH	0.5	4.8E-04	Glutathione MH	0.6	0.00
TNNC1	0.7	1.9E-05	PGZZZZ MNaH	0.5	1.3E-03	PC OO MH.12	0.6	0.14
CTHRC1	0.7	4.5E-06	Epoxyeicosatrienoic acid MNaH	0.5	1.6E-02	PG PG MNH	0.6	0.00
PCSK9	0.7	1.0E-07	Pyroglutamylglycine MH	0.5	1.9E-03	Testosterone glucuronide MH	0.6	0.00
GKN2	0.7	2.6E-05	PG MH	0.5	1.3E-02	PGZZZZZZZZZ MH	0.6	0.01
CDH3	0.7	3.6E-04	Adenosine MH	0.5	1.2E-02	PGZZZZZZ MH	0.6	0.00
FAM216B	0.7	9.6E-03	Uric acid MH	0.5	8.2E-07	HexCer O MH.8	0.6	0.00
NAPSA	0.7	9.4E-06	Panthenol M	0.5	7.2E-04	NAE MH.2	0.6	0.00
SFTPA2	0.7	6.5E-08	Hydroxycistetradecenoyl carnitine MH	0.5	2.8E-03	SM O MH.20	0.6	0.00
FHL2	0.7	2.0E-08	NMethyllysine MH	0.5	1.0E-03	FA MH.6	0.6	0.01
ENSG00000277639	0.7	1.4E-02	Orotic acid MH	0.5	4.1E-03	Ribothymidine MH	0.6	0.02
TGFB3	0.7	4.1E-06	Citrulline MH	0.5	4.4E-04	Docosahexaenoic acid MH	0.6	0.01
GEM	0.7	2.0E-06	Amyl salicylate MH	0.5	1.6E-02	PCZ MH	0.6	0.00
FASN	0.7	3.4E-07	Ribothymidine MH	0.5	6.7E-04	MethoxyHydroxyphenylglycol sulfate MH	0.6	0.01
CXCL14	0.7	2.8E-05	Homocysteine MNH	0.5	1.4E-04	PSZZZZZ MH	0.6	0.01
SPOCK2	0.7	4.8E-03	DLAminooctanoic acid MH	0.5	7.0E-04	ST OS MH	0.6	0.00
EMP2	0.7	1.6E-04	LbetaHomothreonine LCESIQTOF MS CE MH	0.5	1.7E-02	PE MH	0.6	0.00
SULF1	0.7	8.2E-06	LAcetylcarnitine MCl	0.4	8.3E-04	PI O MH.1	0.6	0.05
SSC5D	0.7	1.1E-08	Deoxycytidine MH	0.4	4.4E-03	LTryptophan MH	0.5	0.00
CLXN	0.7	0.0E+00	LysoPE MH	0.4	1.9E-03	PC PC MHCOO	0.5	0.01
MME	0.7	5.5E-07	Trimethylammoniobutanoate M	0.4	3.4E-04	PG O MH.11	0.5	0.00
ANXA3	0.7	5.7E-04	NAcetylornithine MH	0.4	7.2E-04	LPC O MH.5	0.5	0.00
ACADL	0.7	8.3E-06	NAcetylvanilalanine MH	0.4	4.8E-03	FA MH.3	0.5	0.02

RTKN2	0.7	2.1E-04	PIZZZ MH	0.4	4.4E-04	PC OO MH.1	0.5	0.14
SNTN	0.7	2.1E-02	Perillic acid MH	0.4	1.0E-03	PE OPE O MH.2	0.5	0.00
ALDH1A3	0.7	3.6E-05	LFucose MH	0.4	2.0E-03	PGZZZZZZZ MH	0.5	0.01
DNAH9	0.7	1.6E-02	PGZZZZZZZ MH	0.4	1.3E-02	NNDimethylguanosine MH	0.5	0.02
LPCAT1	0.7	1.8E-07	Docosahexaenoic acid MH	0.4	1.1E-03	PI PI MH	0.5	0.03
SUSD2	0.7	3.7E-05	TriHOME MH	0.4	1.3E-03	Cer OOH MH.2	0.5	0.02
NGFR	0.7	5.9E-06	Glucosamine MH	0.4	6.0E-03	LPC MH.4	0.5	0.00
ASPN	0.7	1.6E-07	NCarboxyethylgaminobutyric acid MH	0.4	4.4E-03	PG PG MH	0.5	0.03
SCEL	0.7	3.4E-04	Indoleacetic acid MH	0.4	2.5E-02	PC MH.1	0.5	0.00
SDR16C5	0.7	5.3E-06	PIZZZZ MCl	0.4	4.7E-04	PIPZZZZZ MH	0.5	0.00
FBLN2	0.7	1.8E-07	HODE MH	0.4	1.9E-03	TG MH.15	0.5	0.01
FAM167A	0.7	5.0E-07	transDodecenoylcarnitine MH	0.4	2.8E-03	Succinyladenosine MH	0.5	0.02
CCDC170	0.7	4.0E-03	FTA MH MH	0.4	1.3E-02	Pregnanetriol MH	0.5	0.00
S100A2	0.7	8.9E-03	Uridine triphosphate MH	0.4	6.4E-03	SM O MH.21	0.5	0.06
STXBP6	0.7	1.3E-02	LValine MH	0.4	5.0E-03	PC MH.3	0.5	0.00
MLPH	0.7	2.1E-07	Hydroxyindole MH	0.4	1.2E-02	FA MH.5	0.5	0.03
PLCH2	0.7	9.3E-04	pCresol sulfate MH	0.4	6.5E-03	HexCer OOH MH.5	0.5	0.02
PTGFRN	0.7	1.4E-07	LTryptophan MNH	0.4	1.4E-03	Allopregnanolone MH	0.5	0.01
CCDC17	0.7	2.1E-02	Ecgonine methyl ester MH	0.4	8.5E-04	LPC O MH.2	0.5	0.00
COL1A1	0.7	8.4E-06	Adenosine triphosphate MH	0.4	1.7E-02	PG MH.4	0.5	0.05
UBXN10	0.7	2.4E-03	UTP MH	0.4	1.7E-02	LysoPCZ MH.3	0.5	0.00
DNAH6	0.7	9.5E-03	Hepteneoylglycine MH	0.4	4.7E-04	NAGlySer O MH.1	0.5	0.02
CCDC113	0.7	9.2E-03	cisTetradecenoylcarnitine MH	0.4	3.3E-02	PCZZZZZZ MH	0.5	0.00
HPSE2	0.7	2.4E-08	Pregnanetriol MH	0.4	1.3E-03	HexCer OO MH.1	0.5	0.04
PDLIM4	0.7	1.1E-05	Acetylaminoaminomethyluracil MH	0.4	1.2E-02	Cerd MH.5	0.5	0.00
COL3A1	0.7	6.9E-06	Acetaminophen MH	0.4	1.8E-02	AlphaLinolenoyl ethanolamide MH	0.5	0.02
DAPK2	0.7	2.3E-05	PGZZZZZ MH	0.4	6.4E-04	SMd MH.2	0.5	0.00
FMO5	0.7	1.8E-05	BetaineAldehyde MH	0.4	1.2E-03	FA MH	0.5	0.02
CHST6	0.7	5.0E-04	Sepiapterin MH	0.4	2.8E-03	TG MH.4	0.4	0.14
SERPINF1	0.7	2.1E-07	NADP MH	0.4	4.7E-04	TG MH.8	0.4	0.17
RSPH1	0.7	2.5E-02	Atrazinedesethylhydroxy LCESIITFT MS CE MNH	0.4	2.6E-03	PGZZZZZZZZ MH	0.4	0.09
CDHR4	0.7	1.5E-02	Cholesterol sulfate MH	0.3	4.0E-03	Trimethyluric acid MH	0.4	0.00

CA4	0.7	3.5E-03	Deoxyuridine MH	0.3	9.2E-04	FA MH.7	0.4	0.05
KCNE4	0.7	8.2E-09	Trimethylammoniobutanoic acid MH	0.3	1.6E-04	HexCer O MH.6	0.4	0.00
CDHR3	0.7	1.3E-02	LLactic acid MH	0.3	2.9E-04	TG MH.5	0.4	0.14
PRX	0.7	1.2E-02	Salicyluric betaDglucuronide MH	0.3	2.7E-04	SL OO MH.1	0.4	0.00
TP63	0.7	4.1E-03	Trigonelline MH	0.3	6.4E-03	Arachidonic acid MH	0.4	0.03
CD24	0.7	5.8E-04	Hydroxyhexadecenoylcarnitine MH	0.3	1.6E-01	LysoPCZ MH.2	0.4	0.00
SFTPB	0.7	5.4E-05	NAcetylLlysine MH	0.3	3.9E-03	Vanillactic acid MH	0.4	0.01
DNAH2	0.7	1.4E-02	Glycerophosphocholine MH	0.3	2.2E-04	HexCer OOH MH.8	0.4	0.13
VIPR1	0.7	5.9E-03	Uridine diphosphate MH	0.3	2.2E-02	PC OO MH.7	0.4	0.00

Factor 2

Transcriptomics	Value	FDR	Lipidomics	Value	FDR
EHF	1	3.4E-09	Linoleoyl ethanolamide MH	1.0	0.00
SLC44A4	0.90687259	7.4E-09	SM O MH.11	1.0	0.02
TMC5	0.825390169	6.0E-07	Cer OOH MH.14	0.8	0.86
FOXA1	0.80319332	1.8E-08	Cer O MH	0.8	0.01
TRIM29	0.796017701	1.4E-10	Stearic acid MH	0.8	0.02
IRX3	0.791037372	1.7E-10	MG MH.2	0.8	0.02
CDS1	0.751535091	4.0E-07	PE O MH.3	0.8	0.85
PIFO	0.750102173	1.3E-08	SG OHex MH	0.8	0.05
ELAPOR1	0.747076357	2.8E-06	Cer O MH.2	0.7	0.26
LDLRAD1	0.736927204	2.0E-08	Cer OO MH.3	0.7	0.51
CXCL17	0.732066242	1.2E-06	Eicosapentaenoic acid MH	0.7	0.02
PROM2	0.729175084	9.8E-09	DDDPhytanic acid MH	0.7	0.02
MET	0.723991269	1.0E-08	Cerd MH.4	0.7	0.56

ERBB3	0.7201 62269	5.5E-05	Palmitic acid MH	0.7	0.04
WFDC2	0.7150 42218	2.6E-08	PGZZZZ MH.1	0.7	0.05
TJP3	0.7088 00069	1.0E-06	MG MH	0.7	0.04
CDHR4	0.6967 74612	2.9E-08	NAE MH.2	0.7	0.00
RP1	0.6958 07877	1.1E-07	PCOZZ MH	0.7	0.60
MUC15	0.6934 64343	6.6E-06	AlphaLinolenoyl ethanolamide MH	0.7	0.01
IRF6	0.6910 66884	3.3E-08	PEZPZ MH	0.7	0.49
ENKUR	0.6893 81849	3.5E-09	LysoPCP MH.1	0.7	0.43
FOXJ1	0.6881 65684	2.1E-08	SL OO MH	0.7	0.01
SNTN	0.6861 49451	8.6E-09	SMd MH.1	0.7	0.00
CCDC17	0.6850 46165	6.6E-08	SM O MH.18	0.6	0.14
CFTR	0.6847 56948	4.8E-07	DG MH.2	0.6	0.91
RSPH1	0.6831 96355	1.7E-08	Cer OOH MH.12	0.6	0.16
KLF5	0.6827 8976	2.0E-05	HexCer O MH.2	0.6	0.01
RSPH4A	0.6817 33205	5.2E-09	PE OPE O MH.1	0.6	0.59
ZMYND10	0.6809 8895	5.2E-08	Cer OCer O MH	0.6	0.11
AK7	0.6766 79735	2.8E-08	PEPZZ MH	0.6	0.91
ST6GALNAC1	0.6738 44622	1.2E-08	TG MH.9	0.6	0.91
GRHL2	0.6736 80209	8.5E-08	LPC O MH.3	0.6	0.66
CCDC78	0.6702 01088	5.0E-08	PCZPZ MH	0.6	0.94
DNAH2	0.6682 19778	2.9E-08	PC OO MH.9	0.6	0.02
CAPS	0.6649 44286	1.9E-08	PE MH	0.6	0.06
ENSG00000277639	0.6649 02208	3.1E-08	Cer OCer O MH.2	0.6	0.41
SCNN1B	0.6619 62823	8.3E-04	CAR MH.3	0.6	0.01
BMPR1B	0.6615 97149	3.0E-07	LPalmitoylcarnitine MH	0.6	0.03

EPB41L4B	0.6578 89348	1.4E-06	LysoPCZ MH	0.6	0.74
ODAD2	0.6568 90229	3.7E-09	LysoPE MH	0.6	0.51
SAXO2	0.6568 16951	1.3E-08	Dihomogammalinolenic acid MH	0.6	0.06
FAT2	0.6541 83039	3.8E-11	Cer OOH MH.9	0.6	0.92
KLK11	0.6528 19171	2.8E-05	Arachidonic acid MH	0.6	0.02
SYT8	0.6513 61369	2.0E-10	TG MH.7	0.6	0.83
CELSR1	0.6506 0868	8.7E-07	TG MH.4	0.6	0.85
DNAH6	0.6496 39072	8.6E-09	Nervonic acid MH	0.6	0.29
LRRIQ1	0.6470 31186	9.8E-09	Heptadecanoic acid MH	0.6	0.10
CCDC113	0.6465 574	4.9E-09	Cer O MH.4	0.6	0.01
ABCA13	0.6438 18181	1.4E-08	TG MH.3	0.6	0.89
DNAH10	0.6428 10093	8.4E-08	TG MH.12	0.6	0.97
CFAP251	0.6416 26969	7.6E-09	PC OO MH.12	0.6	0.54
PLPP2	0.6410 95778	2.0E-10	Cer OOH MH.13	0.6	0.62
CFAP52	0.6383 46569	1.3E-08	Cer OOH MH.2	0.6	0.35
CFAP45	0.6366 81232	7.9E-09	Myristic acid MH	0.6	0.36
LRRC23	0.6363 39843	4.0E-09	PEZZZZ MH.2	0.6	0.01
CFAP65	0.6358 46427	7.4E-08	LysoPC MH	0.6	0.92
SCNN1A	0.6346 50326	1.7E-04	Stearoylethanolamide MH	0.6	0.16
DNAH9	0.6334 04356	1.2E-08	SM O MH.10	0.6	0.89
CLDN4	0.6320 16883	6.3E-05	Cerd MH.1	0.6	0.23
ATP2C2	0.6299 12651	6.5E-07	CysteineSsulfate MH	0.6	0.12
S100A14	0.6283 67988	5.0E-06	Behenic acid MH	0.6	0.00
AQP5	0.6255 53332	8.9E-06	Tetracosanoic acid MH	0.6	0.01
MGST1	0.6249 20646	1.3E-06	Alanylisoleucine MH	0.5	0.80

AGR2	0.624791196	2.9E-07	Erucic acid MH	0.5	0.01
DNAH7	0.623412447	3.7E-09	Hexanoylcarnitine MH	0.5	0.35
LRRC46	0.622572825	2.6E-08	Docosahexaenoic acid MH	0.5	0.01
ESRP1	0.620157422	7.3E-06	LPC MHCOO	0.5	0.64
VWA3A	0.61738819	6.4E-07	LPC O MH	0.5	0.61
DLEC1	0.617383536	1.5E-06	SM O MH.3	0.5	0.55
PIGR	0.617195648	5.5E-08	HexCer O MH.1	0.5	0.06
DNAH5	0.61620633	1.4E-06	SE MH.1	0.5	0.31
ODAD1	0.615675407	4.5E-07	Ketocholesterol MH	0.5	0.69
DNAH12	0.611923524	1.5E-07	Dehydrocholesterol MH	0.5	0.90
CLXN	0.611435878	#N/A	NAE MH.5	0.5	0.24
NEK5	0.60696451	1.9E-08	SE MH	0.5	0.49
CFAP157	0.606317982	2.0E-07	PEZZZZ MH	0.5	0.02
PLCH1	0.605035607	3.0E-06	Docosatrienoic acid MH	0.5	0.20
IQANK1	0.603937107	1.5E-07	PSZ MH	0.5	0.12
CLIC6	0.603721121	3.2E-08	LPC MH.5	0.5	0.40
DNAH11	0.601996824	1.0E-06	PE O MH.2	0.5	0.10
STK33	0.60199442	1.4E-08	HexCer OO MH.5	0.5	0.28
TMC4	0.601964575	2.8E-05	Linoleic acid MH	0.5	0.07
ADGRF1	0.601897927	1.8E-04	SM O MH.17	0.5	0.00
CFAP74	0.59938308	8.1E-08	NAE MH.4	0.5	0.94
DUOX1	0.599179848	8.1E-05	SM O MH.12	0.5	0.13
CLDN3	0.599083805	1.2E-05	IsobutyrylLcarnitine MH	0.5	0.04
FAM216B	0.598630562	3.2E-08	Cer OOH MH.10	0.5	0.80
SLC34A2	0.59716604	1.5E-06	SL O MH	0.5	0.14

CEACAM6	0.5942 46211	8.1E-05	CL MH.3	0.5	0.06
ROPN1L	0.5937 53049	1.9E-08	PC MH.5	0.5	0.91
TMEM232	0.5915 45036	1.1E-08	PE P MH	0.5	0.92
HECW2	0.5903 83169	8.1E-08	PEZ MH	0.5	0.88
AGR3	0.5901 27637	4.7E-05	SM O MH.13	0.5	0.06
CFAP57	0.5892 13154	6.2E-09	Hydroxybutyrylcarnitine MNH	0.5	0.73
CFAP70	0.5891 41897	2.3E-06	Trihexosylceramide d MH	0.5	0.02
CFAP46	0.5885 98601	2.1E-07	LysoPCP MH	0.5	0.89
CDHR3	0.5874 98344	9.1E-08	SM O MH.21	0.5	0.64
SCNN1G	0.5872 1949	3.2E-04	PE OO MH.1	0.5	0.11
TACSTD2	0.5867 06343	3.6E-05	TG MH.5	0.5	0.75
ODAD4	0.5864 75491	7.9E-09	Nonadecanoic acid MH	0.5	0.51
